# Supplementary material for: Can GPT-3.5 generate and code discharge summaries?
Source: J Am Med Inform Assoc. 2024 Sep 13;31(10):2284–93. doi: 10.1093/jamia/ocae132 (PMC11413433; doi:10.1093/jamia/ocae132)
Supplement: ocae132_Supplementary_Data [file ocae132_supplementary_data.zip › ocae132_Supplementary_Data/supplementary.pdf]

Supplementary Materials

1 List of Codes Targeted in Generation

E10.3299, E10.3519, E10.3531, E10.3599, E10.52, G43.009, G43.501, G43.919, G43.A0, G43.B0, G43.D0, H35.3110, H35.373, H81.21, H81.399, H81.8X3, H81.91, H81.92, S00.11XA, S00.531A, S00.532A, S00.81XD, S00.93XA, S02.0XXA, S02.113A, S02.119A, S02.31XA, S02.32XA, S02.3XXA, S02.401A, S02.402A, S02.40CA, S02.40DA, S02.40EA, S02.40FA, S02.411A, S02.412A, S02.413A, S02.5XXA, S02.601A, S02.609D, S02.611A, S02.61XA, S02.621A, S02.622A, S02.63XA, S02.652A, S02.66XA, S02.81XA, S02.82XA, S02.8XXA, S06.0X9A, S06.1X0D, S06.2X0A, S06.2X6A, S06.2X7A, S06.300A, S06.339A, S06.359A, S06.5X0D, S06.5X1A, S06.5X7A, S06.5X9D, S06.6X1A, S06.6X6A, S06.890A, S06.9X0S, S06.9X3A, S06.9X9A, T82.03XA, T82.09XA, T82.190A, T82.223A, T82.310A, T82.330A, T82.338A, T82.398D, T82.49XD, T82.594A, T82.6XXA, T82.856A, T82.857A, T82.867A, T82.868S, T84.020A, T84.023A, T84.032A, T84.033A, T84.052A, T84.226A, T84.296A, T84.51XA, T84.53XD, T84.59XA, T84.620D, T84.623A, T84.63XA, T84.7XXA, T84.89XA, T85.02XS, T85.43XA, T85.518A, T85.520A, T85.528A, T85.598A, T85.611A, T85.691A, T85.694A, T85.698A, T85.71XA, T85.72XA, T85.848A, T85.898A, T85.9XXA

2 Prompts for GPT-3.5

2.1 Generic prompt

Write a discharge summary with a detailed hospital course for a patient with these conditions:
(list of conditions)

The patient underwent these procedures:
(list of procedures)

The Discharge Summary should have a word limit of 4000 words. The names of people and locations within the discharge summary should be de-identified. Do not state ICD-10 codes in the main body of the text. For any condition involving a numeric range, state explicitly a number within that range (e.g., for a patient with blood glucose between 7.0 and 11.0 mmol/l generate "patient's glucose level was 8.6 mmol/l"). For conditions with the keyword "other" specify the condition that falls into the set of "other" - e.g., for "Other specified congenital malformations of skin" generate "Aplasia cutis congenita". For conditions with the keyword "unspecified", do not include the word "unspecified" within the main body of text - e.g., for the code H35.00 (Unspecified background retinopathy) generate the statement "the patient suffers from background retinopathy". At the end of the discharge summary add a paragraph with the header "DISCHARGE DIAGNOSES AND PROCEDURES" and assign ICD-10 codes to the discharge diagnoses and procedures, for each concept stating its ICD-10 code and its description (e.g., Essential (Primary) Hypertension [I10]).

2.2 Example prompt

Write a discharge summary with a detailed hospital course for a patient with these conditions:
1. Type 1 diabetes mellitus with proliferative diabetic retinopathy with traction retinal detachment not involving the macula, right eye
2. Non-pressure chronic ulcer of left heel and midfoot with unspecified severity
3. Type 1 diabetes mellitus with diabetic polyneuropathy
4. Type 1 diabetes mellitus with foot ulcer
5. Type 1 diabetes mellitus with proliferative diabetic retinopathy without macular edema, bilateral
6. Long term (current) use of insulin
7. Major depressive disorder, single episode, unspecified
8. Personal history of nicotine dependence
9. Fever, unspecified

The patient underwent these procedures:
1. Fusion of Left Tarsal Joint with Internal Fixation Device, Open Approach
2. Division of Left Foot Tendon, Open Approach
3. Insertion of External Fixation Device into Left Tibia, Percutaneous Approach

The Discharge Summary should have a word limit of 4000 words. The names of people and locations within the discharge summary should be de-identified. Do not state ICD-10 codes in the main body of the text. For any condition involving a numeric range, state explicitly a number within that range (e.g., for a patient with blood glucose between 7.0 and 11.0 mmol/l generate "patient's glucose level was 8.6 mmol/l"). For conditions with the keyword "other" specify the condition that falls into the set of "other" - e.g., for "Other specified congenital malformations of skin" generate "Aplasia cutis congenita". For conditions with the keyword "unspecified", do not include the word "unspecified" within the main body of text - e.g., for the code H35.00 (Unspecified background retinopathy) generate the statement "the patient suffers from background retinopathy". At the end of the discharge summary add a paragraph with the header "DISCHARGE DIAGNOSES AND PROCEDURES" and assign ICD-10 codes to the discharge diagnoses and procedures, for each concept stating its ICD-10 code and its description (e.g., Essential (Primary) Hypertension [I10]). Finish the document with a DISCHARGE STATUS section for the patient - either "DEAD" or "ALIVE"

3 Non-Hierarchical Metrics

For evaluating the model's test performance, we employed standard information retrieval metrics commonly used in LTMC tasks—micro- and macro-averaged Precision (Eq. 4), Recall (Eq. 5), and F1 scores (Eq. 6). These metrics rely on the true positives (Eq. 1), false positives (Eq. 2), and false negatives (Eq. 3),

derived from the prediction (Z_d) and gold standard (Y_d) sets for each document (d) in the evaluation set (D).

$$TP_d = |Z_d \cap Y_d| \tag{1}$$

$$FP_d = |Z_d - Y_d| \tag{2}$$

$$FN_d = |Y_d - Z_d| \tag{3}$$

$$P = \frac{\sum_{d=1}^{D} TP_d}{\sum_{d=1}^{D} TP_d + FP_d} \tag{4}$$

$$R = \frac{\sum_{d=1}^{D} TP_d}{\sum_{d=1}^{D} TP_d + FN_d} \tag{5}$$

$$F_1 = \frac{2 \cdot P \cdot R}{P + R} \tag{6}$$

Micro-averaging (*Eq.*7) assigns equal weight to each prediction, favouring high-population classes (*e.g.,* hypertension). Macro-averaging (*Eq.*8), in contrast, computes the performance for each unique label and averages across the label space, giving each label's average result equal weight regardless of their population. This highlights poor performance in less common classes. Our primary evaluation metrics common with the majority of previous work are micro-F1 (*Eq.*9) and macro-F1 scores (*Eq.*10)

$$M_{micro} = M\left(\sum_{l=1}^{|L|} TP_l, \sum_{l=1}^{|L|} FP_l, \sum_{l=1}^{|L|} FN_l\right) \tag{7}$$

$$M_{macro} = \frac{1}{|L|}\sum_{l=1}^{|L|} M(TP_l, FP_l, FN_l) \tag{8}$$

$$F_{1(micro)} = \frac{2 \cdot P_{micro} \cdot R_{micro}}{P_{micro} + R_{micro}} \tag{9}$$

$$F_{1(macro)} = \frac{2 \cdot P_{macro} \cdot R_{macro}}{P_{macro} + R_{macro}} \tag{10}$$

4 Hierarchical Metrics

4.1 Set-Based

Let X represent a set of N' ICD codes individually denoted as x_1, x_2, ... $x_{N'}$. Let $AN_j(x_i)$ denote a function that returns a set of all ancestors of the code x_i up to the depth j within the ontology. The augmented set $X_{(aug)}$ consists of the union of the original codes in X and their ancestors *Eq.*11.

$$X_{(aug)} = X \cup \{AN_j(x_i)|1, ..., N'\} \tag{11}$$

In set-based hierarchical evaluation we calculate the Precision, Recall and F1 score with their standard formulae based on re-defined TP(*Eq.*12), FP(*Eq.*13), and FN(*Eq.*14).

$$TP_{(set)d} = |Z_{(aug)d} \cap Y_{(aug)d}| \tag{12}$$

$$FP_{(set)d} = |Z_{(aug)d} - Y_{(aug)d}| \tag{13}$$

$$FN_{(set)d} = |Y_{(aug)d} - Z_{(aug)d}| \tag{14}$$

4.2 CoPHE

In CoPHE the ancestor labels are associated with the number of descendant codes present in the respective sets. This is handled by re-defining TP, FP, and FN per document (d) per code family (c) (*Eq.* 15, 16, 17). The per-document value of TP, FP, and FN is the sum of the respective per-code-family metrics (example for TP in *Eq.* 18). The $F1$ scores for CoPHE are then calculated based on the precision and recall calculated from the CoPHE versions of TP, FP, and FN. Preserving counts allows tracking whether the correct number of descendants were predicted, or whether there is under-/over-predictions (e.g., three different instances of heart disease predicted when only one was expected).

$$TP_{(CoPHE)c,d} = min(|Z_{(aug)c,d}|, |Y_{(aug)c,d}|) \tag{15}$$

$$FP_{(CoPHE)c,d} = max(|Z_{(aug)c,d}| - |Y_{(aug)c,d}|, 0) \tag{16}$$

$$FN_{(CoPHE)c,d} = max(|Y_{(aug)c,d}| - |Z_{(aug)c,d}|, 0) \tag{17}$$

$$TP_{(CoPHE)d} = \sum_{c=1}^{|C|} TP_{(CoPHE)c,d} \tag{18}$$
